# Features of Methylation and Gene Expression in the Promoter-Associated CpG Islands Using Human Methylome Data

**DOI:** 10.1155/2012/598987

**Published:** 2012-03-04

**Authors:** Xin Du, Leng Han, An-Yuan Guo, Zhongming Zhao

**Affiliations:** ^1^College of Life Sciences, Central China Normal University, Wuhan 430079, China; ^2^Department of Biomedical Informatics, Vanderbilt University School of Medicine, Nashville, TN 37232, USA; ^3^Departments of Radiology and Medicine, Stanford University School of Medicine, Stanford, CA 94305, USA; ^4^Hubei Bioinformatics & Molecular Imaging Key Laboratory, Department of Systems Biology, College of Life Science and Technology, Huazhong University of Science and Technology, Wuhan 430074, China; ^5^Department of Psychiatry, Vanderbilt University School of Medicine, Nashville, TN 37232, USA; ^6^Department of Cancer Biology, Vanderbilt University School of Medicine, Nashville, TN 37232, USA

## Abstract

CpG islands are typically located in the 5′ end of genes and considered as gene markers because they play important roles in gene regulation via epigenetic change. In this study, we compared the features of CpG islands identified by several major algorithms by setting the parameter cutoff values in order to obtain a similar number of CpG islands in a genome. This approach allows us to systematically compare the methylation and gene expression patterns in the identified CpG islands. We found that Takai and Jones' algorithm tends to identify longer CpG islands but with weaker CpG island features (e.g., lower GC content and lower ratio of the observed over expected CpGs) and higher methylation level. Conversely, the CpG clusters identified by Hackenberg et al.'s algorithm using stringent criteria are shorter and have stronger features and lower methylation level. In addition, we used the genome-wide base-resolution methylation profile in two cell lines to show that genes with a lower methylation level at the promoter-associated CpG islands tend to express in more tissues and have stronger expression. Our results validated that the DNA methylation of promoter-associated CpG islands suppresses gene expression at the genome level.

## 1. Introduction

CpG islands (CGIs), which are clusters of CpG dinucleotides in GC-rich regions, are often located in the 5′ end of genes and are considered as gene markers in vertebrate genomes [1–3]. These CpG islands, especially promoter-associated CpG islands, play important roles in gene silencing, genomic imprinting, X-chromosome inactivation, and tumorigenesis [4]. Due to the functional importance of CpG islands in transcriptional regulation and epigenetic modifications [5], multiple algorithms have been developed to identify CpG islands in a genome or a specific sequence. Overall, these algorithms can be classified into two groups: traditional algorithms and new algorithms. Traditional algorithms are based on three features and parameters (length, GC content, and ratio of the observed over the expected CpGs (CpG O/E)) [2, 4, 6, 7], while new algorithms are based on statistical property [8, 9]. Substantial debate exists as to which algorithm performs better and in which context, such as in organisms, tissues, or developmental stages [4, 8, 10–12]. Comparing different features of CpG islands, especially length of the predicted islands [11], our previous study suggested that Takai and Jones' algorithm is more appropriate overall for identifying promoter-associated islands of CpGs in vertebrate genomes [10]. However, the major biological patterns would remain similar regardless of the algorithm being used [13, 14]. For example, the density of CpG islands is highly correlated with the number or the size of the chromosomes in mammalian genomes [13], and the number of CpG islands varies greatly among fish genomes [14]. Nevertheless, the recent study by Hackenberg et al. showed that setting the *P* value to 10^−20^ could largely improve the performance [11]. Considering the information above, a further comparison using a similar number of CpG islands identified by different algorithms might provide us additional insights into biological features and their regulation in the cellular system.

DNA methylation is an important epigenetic modification at the transcriptional regulation level, and this process is directly and substantially related to CpG islands [5, 15, 16]. Over 50% of human genes are associated with CpG islands in their promoter regions [3, 7], while ~15–35% of CpG islands are located in the promoter regions of genes, according to several algorithms [10, 11]. Promoter-associated CpG islands have different features compared to other types of CpG islands; these features include a longer CpG island sequence, higher GC content, and higher CpG O/E ratio [6, 10]. However, the correlation between the methylation of promoter-associated CpG islands and gene expression is more complex than what investigators previously thought. Several studies reported that the methylation level of promoter-associated CpG islands is negatively correlated with gene expression strength [16, 17] while others observed no or weak correlations [18, 19]. This difference may be due to the dynamic and complex nature of methylation in cellular systems [20–23]. Until 2009, when single-base resolution methylome data was released, studies regarding the relationship between CpG islands, methylation, and gene expression were limited. Additionally, the earlier data was often at the computational level and used features based on low-resolution data generated by array-based technology [24]. In this study, we performed an extensive investigation of the correlations between CpG islands, methylation, and gene expression by taking advantage of newly available whole genome base-resolution methylation profiling [25].

## 2. Materials and Methods

### 2.1. Identification of CpG Islands in the Human Genome

We used the stringent criteria presented in Takai and Jones [4] to search CpG islands: length ≥ 500 bp, GC content ≥ 55%, and CpG O/E ratio ≥ 0.65. We also used the CpG cluster algorithm developed by Hackenberg et al. [8] to identify CpG clusters. In this study, we integrated the use of both CpG islands and CpG clusters because of their expected similarities in terms of their service as gene markers and measurement of methylation status. To identify comparable numbers of CpG clusters and CpG islands in the whole genome, we used median distance of each as the cutoff value and the *P* value < 10^−15^ or < 10^−20^, respectively. These two *P* value cutoffs allowed us to obtain two sets of CpG clusters for comparison. We then downloaded CpG islands annotated by the UCSC Genome Browser (http://genome.ucsc.edu/), which were screened from the human genome by the following criteria: length > 200 bp; GC content ≥ 50%, and CpG O/E ratio > 0.6. Moreover, the UCSC algorithm searches the reference sequence one base at a time, scores each dinucleotide (+17 for CpG and −1 for others), and identifies maximally scored segments.

### 2.2. Sequence Data and Gene Annotation

We downloaded the assembled human genome sequence from the National Center for Biotechnology Information (NCBI, build 36, ftp://ftp.ncbi.nih.gov/genomes/). We also extracted the Transcriptional Start Sites (TSS) for human Refseq genes from the UCSC Genome Browser (http://genome.ucsc.edu/). The promoter region was defined as −1,500 to +500 bp around the TSS, as previously described [10].

### 2.3. Base-Resolution Methylation Data in the Human Genome

To evaluate the methylation status in CpG islands and CpG clusters, we downloaded the base-resolution methylation data for H1 and IMR90 cell lines in the human genome reported in Lister et al. [25], which are the first human DNA methylomes at base resolution (http://neomorph.salk.edu/human_methylome/data.html). Here, we used methylation broadness [26] to evaluate the methylation level in a CpG island. The methylation broadness represents the fraction of cytosine sites detected as methylated in a given DNA segment. It can be calculated as the proportion of methylated CpG sites over the total sites in a sequence (i.e., number of methylated CpG sites/total CpG sites) [26]. A methylated CpG site is defined as the CpG site with at least one methylated read. Additionally, we obtained the methylation data in the promoter regions in the human H1 cell line from Bock et al. [27].

### 2.4. Gene Expression in the Human Genome

Human gene expression data in the second version of the Gene Expression Atlas reported in Su et al. [28] was directly obtained from the author Andrew Su. There were 79 human tissues studied. Defining the expression of a gene in a specific tissue was described in previous work [29, 30]. Briefly, the average difference (AD) value was required at least 200. Then, each gene was classified according to one of four groups: (1) housekeeping genes (expressed in all 79 tissues), (2) widely expressed genes (expressed in more than 80% but less than 100% of tissues), (3) moderately expressed genes (expressed in 20%–80% of tissues), and (4) narrowly expressed genes (expressed in less than 20% of genes) [31]. The expression strength was calculated by the average expression value among all 79 tissues followed by the logarithm transformation. Furthermore, we obtained separate gene expression datasets that are specifically for the human H1 and IMR90 cell lines from Lister et al. [25] and a gene expression dataset for H1 cell line from Bock et al. [27].

## 3. Results and Discussion

### 3.1. Features of CpG Islands Identified by Different Algorithms

Our previous studies showed that the number of CpG islands identified by the different algorithms (e.g., Gardiner-Garden and Frommer (1987) [2], Takai and Jones (2002) [4], and CpG clusters [8]) varies greatly, either in the human genome or other vertebrate genomes [10, 13, 14]. Particularly, CpG clusters have several unique features (e.g., the greater number of CpG clusters identified in the human genome) that are largely dependent on the *P* value assigned and the length cutoff value [11]. To attain a comprehensive comparison of the performances of the major existing algorithms, in this study, our design utilized the parameters of these key algorithms so that the number of CpG islands (or clusters) would be similar and then applied this strategy to the human genome. The underlying rationale is that methylation regulation often lies in the promoter regions of genes where CpG islands are representative. Accordingly, we assigned *P* value 10^−15^ as the cutoff to identify 37,184 CpG clusters, which is close to the number of CpG islands (37,729) identified by Takai and Jones' algorithm. Moreover, we assigned the *P* value 10^−20^ as the cutoff to identify 25,454 CpG clusters, and this number is similar to the one identified by the UCSC Genome Browser (27,639) (Table 1). To save space, we abbreviated the CpG islands identified by Takai and Jones' algorithm as TJ-CGIs, CpG islands identified by the USCS Genome Browser as UCSC-CGIs, CpG clusters by *P* value cutoff 10^−15^ as CGCs-15, and CpG clusters by *P* value cutoff 10^−20^ as CGCs-20. We further examined how the CpG clusters were associated with the promoter regions after applying these stringent *P* value cutoffs. Remarkably, the proportion of promoter-associated CpG clusters increased dramatically: 38.8% of all CGCs-15 and 47.6% of all CGCs-20 were promoter associated, but only 14.7% displayed association by using the default cutoff value [10]. This increase was similarly shown in Hackenberg et al. [11]. Therefore, we could obtain not only a similar number of CpG islands/clusters but also more promoter-associated CpG islands/clusters for a systematic investigation of the features of CpG islands/clusters, methylation, and gene expression in the human genome.

**Figure 1:**
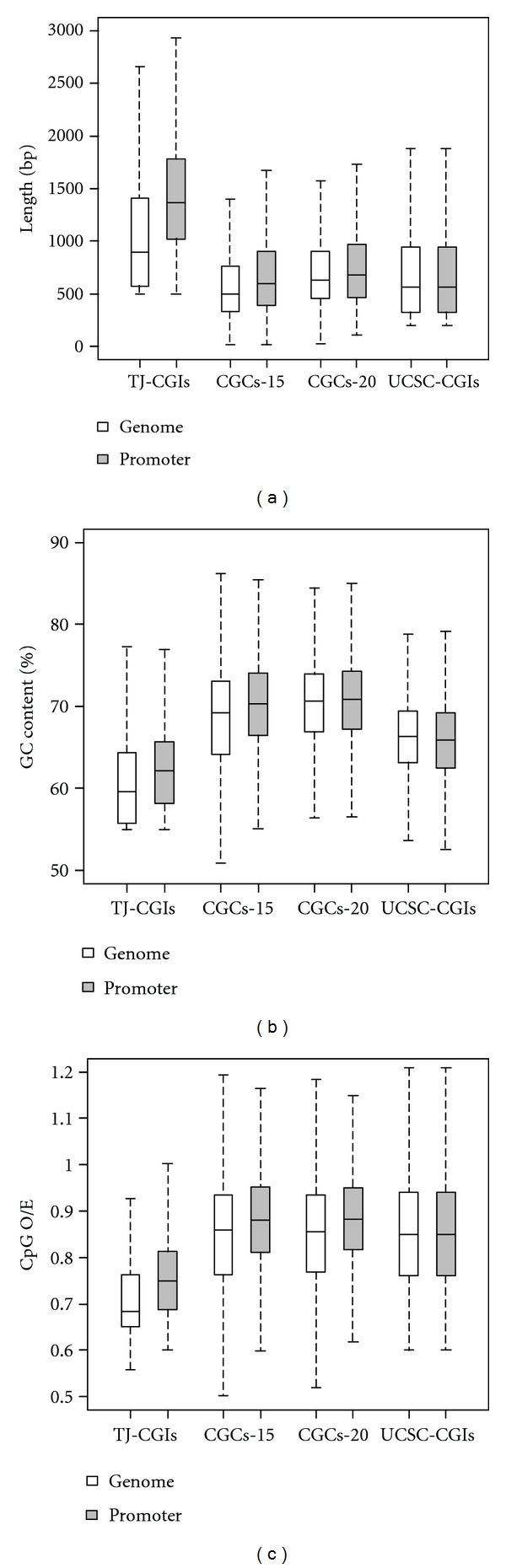
Distribution of features of CpG islands identified by different algorithms. (a) Length. (b) GC content (%). (c) CpG O/E.

The average length of TJ-CGIs was 1089 bp, which was much longer than the average length of CGCs-15 (605 bp), CGCs-20 (727 bp), and UCSC-CGIs (763 bp) (Table 1). Conversely, TJ-CGIs had weaker CpG island features. For example, they had a lower GC content (60.6%) and a lower CpG O/E ratio (0.717) than those of CGCs-15 (GC content = 68.7%, CpG O/E = 0.855), CGCs-20 (GC content = 70.3%, CpG O/E = 0.853), and UCSC-CGIs (GC content = 66.1%, CpG O/E = 0.862) (Table 1). In addition, we used box plot (with the values for maximum, minimum, median, 75% quantile, and 25% quantile) to display the distribution and features of CpG islands identified by each algorithm (Figure 1). Overall, the difference was largely due to the Hackenberg CpG cluster algorithm's tendency to identify short regions with strong CpG island features [8, 10]. This comparison indicated that features of CpG islands or clusters relied on their length—the longer they are, the weaker the features they would have.

CpG islands are often located in the 5′ end of genes; thus, they are considered to be gene markers [3, 32]. Our previous studies showed that the features of CpG islands are different in the promoter and other regions [13, 31]. Therefore, the features of promoter-associated CpG islands are more important than other genomic regions when evaluating the performance of differing CpG island/cluster algorithms. Among the 37,729 TJ-CGIs, we found 13,270 (35.0%) mapped to the promoter regions of 12,521 known genes and, on average, 1.05 TJ-CGIs per gene. Similarly, we found 14,419 (38.8%) of CGCs-15 mapped to 11,292 genes and, on average, 1.28 CGCs per gene, 12,115 (47.6%) of CGCs-20 mapped to 10,245 genes and, on average, 1.18 CGCs per gene, and 12,297 (44.5%) of UCSC-CGIs mapped to 11,744 genes and, on average, 1.05 UCSC-CGIs per gene (Table 1). We observed more CpG clusters than TJ-CGIs and UCSC-CGIs in the promoter regions of genes. One main reason for this finding is that multiple CpG clusters are more likely to be identified within the same promoter region than TJ and UCSC algorithms, and several CpG clusters are often embedded within one single CpG island [10, 11].

While the total number of CpG islands or CpG clusters varied among the four algorithms (ranging from 25,454 CGCs-20 to 37,729 TG-CGIs, 1.48-fold difference, Table 1), the number of promoter-associated CpG islands identified by these algorithms was actually similar (ranging from 12,115 CGCs-20 to 14,419 CGCs-15, 1.19-fold difference), confirming that CpG islands are the most important features in the promoter regions. Notably, there would be approximately 5.3-fold difference between the number of TJ-CGIs and CpG clusters when the default parameters are used in these algorithms [10]. The proportion of promoter-associated CpG islands was also very similar, that is, in a range of 35.0% (TJ-CGIs) to 47.6% (CGCs-20), 1.36-fold difference (Table 1). Compared to the features of CpG islands at the whole genome level, the promoter-associated CpG islands showed stronger features, for example, increased length, higher GC content, and larger CpG O/E ratio (Figure 1). These observations are consistent with the previous studies [6, 31], but here, we focused on a specific group of CpG clusters identified by CpG cluster algorithms using stringent criteria that are comparable to a CpG island search.

### 3.2. Methylation Status of CpG Islands or Clusters

The methylation level varies within CpG islands [11]. Bock et al. [33] suggested that it should be sufficient to measure average methylation level rather than assaying every single CpG dinucleotide in a genomic region. Considering that suggestion, it would be interesting to evaluate methylation at the whole CpG island or cluster level using the highest resolution methylation data, that is, the base-resolution human methylome data, as previous studies largely relied on limited microarray-based low resolution methylation data or computational prediction [24]. Here, we applied methylation broadness, which we recently proposed and was described in Su et al. [26] to evaluate the methylation level in a CpG island or cluster. The methylation broadness is calculated as the proportion of methylated CpG sites over the total sites in a sequence (i.e., number of methylated CpG sites/total CpG sites) [26]. Since the methylation status is dynamic among different types of cells, we evaluated the methylation level in both the H1 and IMR90 cell lines in the human genome.

**Figure 2:**
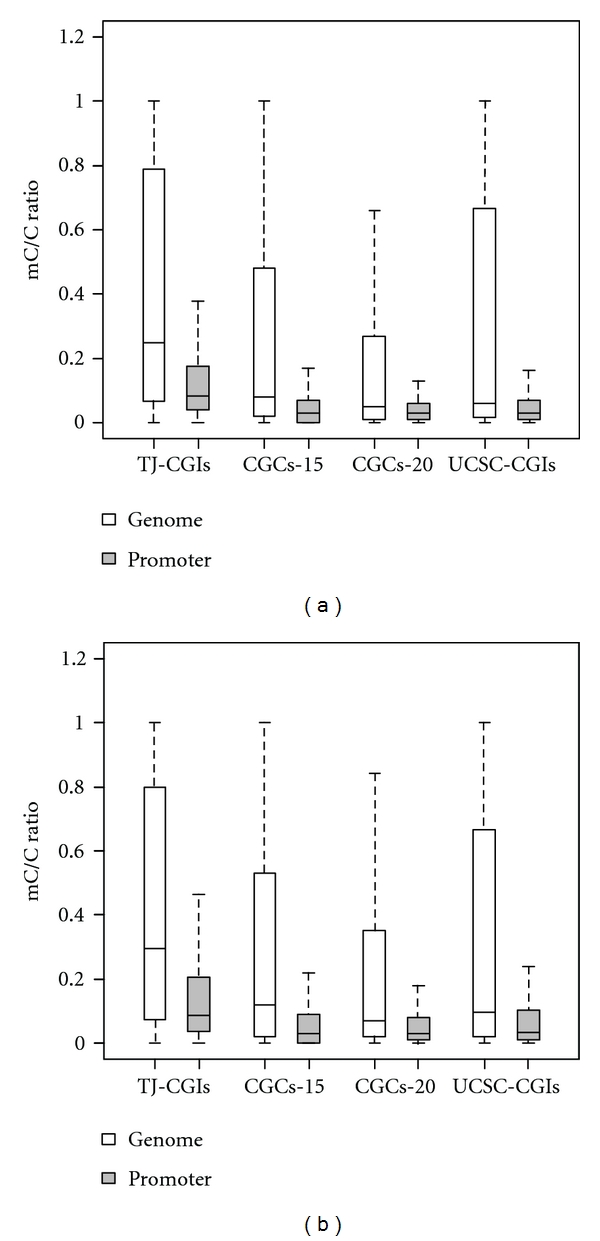
Methylation level of the CpG islands or clusters in the whole genomic regions or associated with the promoter regions. (a) Methylation data was based on human methylome from H1 cell line. (b) Methylation data was based on human methylome from IMR90 cell line.

At the genome level, our results showed that TJ-CGIs had a higher average methylation level (mC/C ratio = 0.403) than CGCs-15 (mC/C ratio = 0.266), CGCs-20 (mC/C ratio = 0.205), and UCSC-CGIs (mC/C ratio = 0.297) in the H1 cell line. Here, mC/C denotes the ratio of methylated over unmethylated nucleotides C at the CpG sites. A similar pattern could be observed in the IMR90 cell line, as shown in Figure 2.

Promoter-associated CpG islands are thought to be mostly unmethylated or to maintain a low methylation level [18]. Compared to CpG islands across the whole genome, the methylation level of promoter-associated CpG islands decreased dramatically. In the human H1 cell line, promoter-associated TJ-CGIs had, on average, an mC/C ratio 0.177, while this ratio was 0.100 for CGCs-15, 0.084 for CGCs-20, and 0.108 for UCSC-CGIs (Figure 2(a)). Similar to our observation of the methylation pattern in CpG islands or clusters across the whole genome, promoter-associated TJ-CGIs had the highest average methylation level measured by mC/C ratio, while CpG islands or clusters identified by the other three algorithms had similar methylation levels. This difference could be related to the fact that TJ-CGIs cover longer genomic regions, some of which bear a higher methylation level. We observed a similar pattern when the methylation data in the IMR90 cell line was used (Figure 2(b)). By this measurement, our comparison suggested that the algorithm using the assigned *P* value cutoff 10^−20^ in Hackenberg et al. [8] had the best performance to identify CpG islands or clusters with a low methylation level.

### 3.3. Methylation of Promoter-Associated CpG Islands and Gene Expression

Methylation plays an important role in the regulation of gene expression [18, 34]. Previous studies showed that DNA methylation typically represses gene expression [15, 16]. Lister et al. [25] first displayed the correlation between the gene body methylation level and gene expression strength at the single base resolution. Here, we further analyzed the correlation between the methylation level of promoter-associated CpG islands and genes expression strength based on Lister et al. data [25]. For those genes having both expression data and methylation data in their promoter-associated CpG islands, we found a very weak negative correlation (Table 2). Moreover, we calculated Pearson's correlation coefficient between methylation of promoters and gene expression strength using Bock et al.'s [27] expression profile and methylation profile in the promoter regions across the genome in the human H1 cell line. We found a weak negative correlation between gene expression strength and methylation of promoter regions in the H1 cell line (Pearson correlation coefficient = −0.242, *P* < 2.2 × 10^−16^). One possible reason for the observation of this weak correlation is that some promoter-associated CpG islands, or some promoters with methylation levels across different tissues, are negatively correlated with gene expression strength, while some others are positively correlated [35]. Following this observation, we investigated the correlation between the expression strength and methylation of promoter-associated CpG islands according to different genes categorized by their tissue expression.

**Figure 3:**
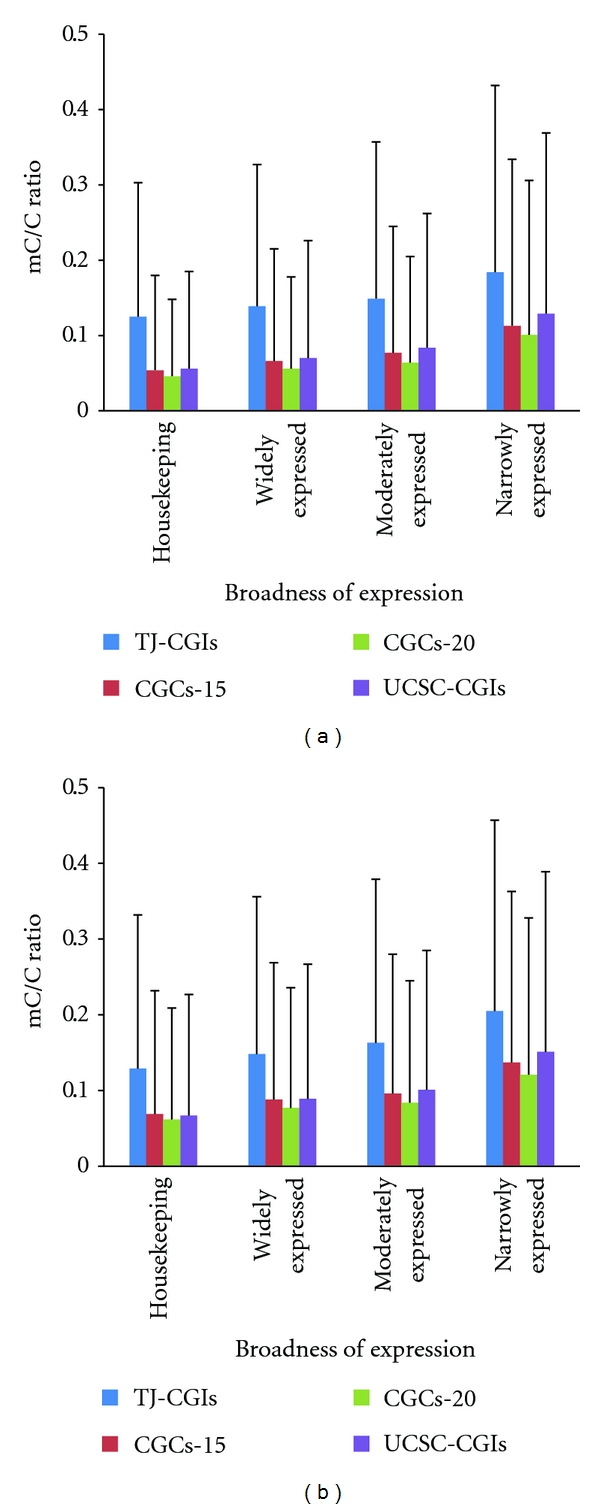
Relationship between methylation level of promoter-associated CpG islands and the number of expressed tissues. (a) H1 cell line. (b) IMR90 cell line.

We first investigated the correlation between promoter-associated CpG islands and broadness of expression. In the human H1 cell line, the average methylation level in promoter-associated TJ-CGIs in housekeeping genes was 0.125, compared to 0.139, 0.149, 0.184 in widely expressed genes, moderately expressed genes, and narrowly expressed genes, respectively (Figure 3(a)). The methylation level of CGCs-15, CGCs-20, and UCSC-CGIs was much lower than in that of TJ-CGIs. For example, the average methylation level in CGCs-15 in housekeeping genes, widely expressed genes, moderately expressed genes, and narrowly expressed genes was 0.054, 0.066, 0.077, and 0.113, respectively (Figure 3(a)). Overall, at the genome level, there was a trend that genes whose methylation level at the promoter-associated CpG islands or clusters was lower tended to express in more tissues, regardless of which specific algorithm was used. Aside from the pluripotent H1 cell line, we also examined the methylation level in the IMR90 cell line, and the conclusion remained the same (Figure 3(b)).

**Figure 4:**
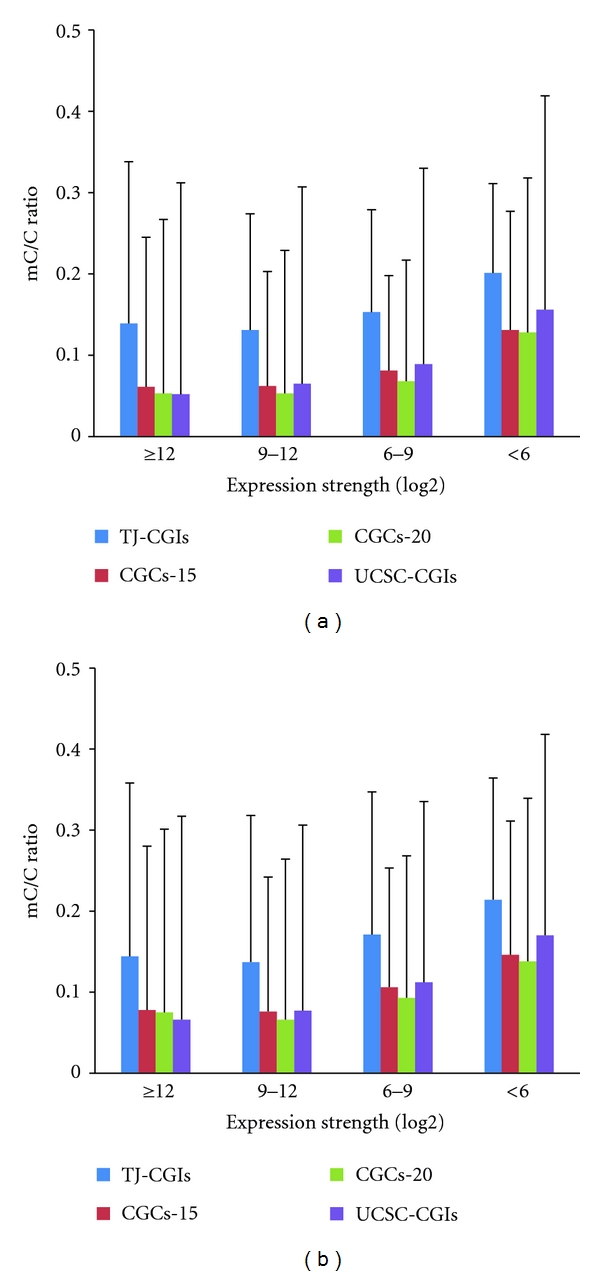
Relationship between methylation level of promoter-associated CpG islands and expression strength. (a) Methylation data was based on human methylome from H1 cell line. (b) Methylation data was based on human methylome from IMR90 cell line.

**Figure 5:**
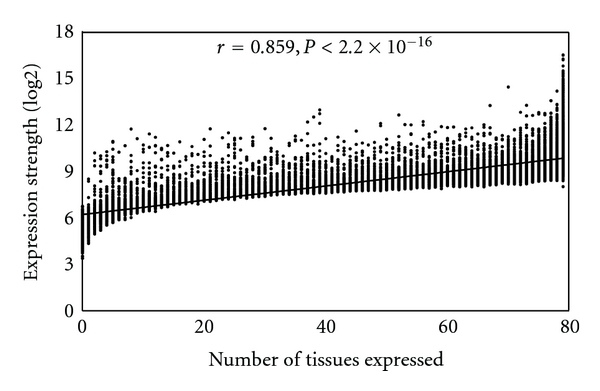
Correlation between the number of expressed tissues and expression strength.

To further study the correlation between the methylation of promoter-associated CpG islands and gene expression strength, we used the average expression level across the 79 human tissues in the second version of the Gene Atlas dataset to represent the expression strength of a gene. In the human H1 cell line, the methylation level of the promoter-associated CpG islands in the genes with a strong expression strength (log2 expression value > 9) was much lower than those with a weak expression strength (log2 expression value < 6) (Figure 4(a)). One should note that the methylation level of promoter-associated CpG islands in genes whose expression strength values are in the range of 9–12 is similar to that of genes whose expression strength values are greater than 12. Again, a similar trend was observed when we used the methylation data from the IMR90 cell line (Figure 4(b)). Furthermore, we found a significant positive correlation between broadness of expression and expression strength (Pearson correlation *r* = 0.859, *P* < .2 × 10^−16^, Figure 5). This finding explained the consistency between broadness of expression and strength of expression.

In summary, genes with a lower methylation level at the promoter-associated CpG islands tend to express in more tissues and have stronger expression strength, while genes with a higher methylation level at the promoter-associated CpG islands tend to express in fewer tissues and have weaker expression strength.

## 4. Conclusion

In this study, we systematically investigated the features of CpG islands or clusters identified by several major algorithms in the human genome, taking advantage of recently released single-base human methylome and gene expression Atlas datasets. Because many more CpG clusters were previously found compared to the number of genes, in this study, we applied stringent criteria to generate a comparable number of CpG islands identified by the traditional Takai and Jones algorithm, or a similar number of protein-coding genes in the human genome. Our results show that Takai and Jones' algorithm tends to identify longer CpG islands, yet weaker CpG island features as well as a higher methylation level. However, this algorithm typically identifies one unique promoter-associated CpG island for a gene. Conversely, Hackenberg et al.'s algorithm is likely to identify multiple promoter-associated CpG clusters for a gene, but its CpG clusters tend to have stronger CpG island features, such as a higher GC content, higher CpG O/E ratio, and lower methylation level. This comparative study indicated that, with the appropriate, stringent cutoff value, we may identify CpG clusters that are more representative of the gene markers by uniquely mapping to the promoter regions of genes, maintaining a low methylation level, and strongly correlating gene expression among tissues. These CpG clusters may be more functional among all the CpG clusters and would be identified by the default parameters; thus, we may denote them as core CpG clusters.

Although DNA methylation has been widely thought to suppress gene expression, largely through its methylation regulation at the gene's promoter region [15, 16], we found only a weak correlation between DNA methylation and gene expression strength across the whole human genome. Our results based on the gene expression broadness categories (housekeeping, widely expressed, moderately expressed, and narrowly expressed) showed that genes with a lower methylation level at the promoter-associated CpG islands tend to be expressed in more tissues and have stronger expression strength. Our results validated that the DNA methylation of promoter-associated CpG islands suppresses gene expression.

In this study, we did not take into account the missing data in the methylomes. That is, if a CpG site was not sequenced, it might be implicated as unmethylated. According to Lister et al. [25], their methylome sequencing covered 94% of the cytosines in the human genome. Considering that sequencing of gene regions has generally more coverage and higher quality than noncoding regions and our measure is the broadness of methylation in the gene regions, the effect of data missingness on our conclusions is expected to be minor. Furthermore, we applied the broadness measurement to assess methylation level in a genomic region. In this measurement, methylation of each CpG site is either methylated or unmethylated. It would be more informative by taking into account the extent of methylation at each CpG site, as such data is available from next generation sequencing. We recently proposed a deepness measurement [26], which can be combined with the broadness measurement in our future analysis.

## Figures and Tables

**Table 1:** Summary of the CpG islands (CGIs) or CpG clusters (CGCs) identified by different algorithms in the human genome.

Method*	Whole genome CGIs					Promoter-associated CGIs						
	# CGIs	Length (bp)	GC content (%)	CpG O/E**		# CGIs	Proportion (%)	# genes	CGIs/gene	Length (bp)	GC content (%)	CpG O/E**
TJ-CGIs	37, 729	1089	60.6	0.717		13,207	35.0	12, 521	1.05	1477	62.2	0.759
CGCs-15	37, 184	605	68.7	0.855		14,419	38.8	11, 292	1.28	694	70.1	0.885
CGCs-20	25, 454	727	70.3	0.853		12,115	47.6	10, 245	1.18	767	70.6	0.885
UCSC-CGIs	27, 639	763	66.1	0.862		12,297	44.5	11, 744	1.05	964	69.3	0.862

*The methods/algorithms for screening CpG islands or CpG clusters are described in Section 2.

**CpG O/E: the ratio of the observed versus expected number of CpG dinucleotides in a sequence.

**Table 2:** Pearson's correlation coefficient between methylation level of promoter-associated CGIs and gene expression strength.

Method*	H1			IMR90	
	*r*	*P*		*r*	*P*
TJ-CGIs	−0.016	0.128		−0.021	0.0419
CGCs-15	−0.037	4.78 × 10^−4^		−0.036	7.52 × 10^−4^
CGCs-20	−0.037	9.47 × 10^−4^		−0.040	3.33 × 10^−4^
UCSC-CGIs	−0.045	1.40 × 10^−5^		−0.038	2.89 × 10^−4^

*The methods/algorithms for screening CpG islands or CpG clusters are described in Section 2.
